# The #VaccinesWork Hashtag on Twitter in the Context of the COVID-19 Pandemic: Network Analysis

**DOI:** 10.2196/38153

**Published:** 2022-10-28

**Authors:** Aïna Fuster-Casanovas, Ronnie Das, Josep Vidal-Alaball, Francesc Lopez Segui, Wasim Ahmed

**Affiliations:** 1 Unitat de Suport a la Recerca de la Catalunya Central Fundació Institut Universitari per a la Recerca a l'Atenció Primària de Salut Jordi Gol i Gurina Sant Fruitós de Bages Spain; 2 Health Promotion in Rural Areas Research Group Gerència Territorial de la Catalunya Central, Institut Català de la Salut Sant Fruitós de Bages Spain; 3 Audencia Business School Nantes France; 4 Faculty of Medicine University of Vic-Central University of Catalonia Vic Spain; 5 Germans Trias i Pujol Hospital Institut Català de la Salut Badalona Spain; 6 Research Group on Innovation Health Economics and Digital Transformation (Institut Germans Trias i Pujol) Badalona Spain; 7 Management School University of Stirling Stirling United Kingdom

**Keywords:** Twitter, social media, COVID-19, misinformation, vaccination, public health, vaccine hesitancy, infodemiology, health campaign, content analysis, social network, layout algorithm

## Abstract

**Background:**

Vaccination is one of the most successful public health interventions for the prevention of COVID-19. Toward the end of April 2021, UNICEF (United Nations International Children’s Emergency Fund), alongside other organizations, were promoting the hashtag #VaccinesWork.

**Objective:**

The aim of this paper is to analyze the #VaccinesWork hashtag on Twitter in the context of the COVID-19 pandemic, analyzing the main messages shared and the organizations involved.

**Methods:**

The data set used in this study consists of 11,085 tweets containing the #VaccinesWork hashtag from the 29th to the 30th of April 2021. The data set includes tweets that may not have the hashtag but were replies or mentions in those tweets. The data were retrieved using NodeXL, and the network graph was laid out using the Harel-Koren fast multiscale layout algorithm.

**Results:**

The study found that organizations such as the World Health Organization, UNICEF, and Gavi were the key opinion leaders and had a big influence on the spread of information among users. Furthermore, the most shared URLs belonged to academic journals with a high impact factor. Provaccination users had other vaccination-promoting hashtags in common, not only in the COVID-19 scenario.

**Conclusions:**

This study investigated the discussions surrounding the #VaccinesWork hashtag. Social media networks containing conspiracy theories tend to contain dubious accounts leading the discussions and are often linked to unverified information. This kind of analysis can be useful to detect the optimal moment for launching health campaigns on Twitter.

## Introduction

The outbreak of the COVID-19 pandemic in December 2020 in China and its rapid spread around the world has highlighted health and health care systems as one of the most important human vulnerabilities. To tackle a virus with a worldwide high transmission rate, institutions identified priorities for combating it; limiting the spread of the virus, providing medical equipment, research, and tackling the sociodemographic consequences were their main objectives [1]. On January 30, 2020, the World Health Organization (WHO) Emergency Committee declared a global health emergency because of rising case reporting rates [2].

In the absence of a vaccine or treatments, social distancing and handwashing were the first measures promoted to reduce the spread of the virus. Next, as a complementary measure, the mass lockdown of the population helped reduce the increase in cases and gave the scientific community time to develop other mechanisms to curb the contagion [3]. In parallel, the development of a vaccine was a key objective for all countries [4]. On December 23, 2020, the European Medicines Agency approved the first vaccine from the Pfizer-BioNTech Comirnaty laboratory [5]. This was followed by the approval of other vaccines, such as the Moderna/Lonza-Spikevax, the Oxford/AstraZeneca, and finally, the Johnson and Johnson/Janssen vaccines, to achieve group immunity as quickly as possible.

Vaccination is one of the most successful public health interventions for the prevention of communicable infectious diseases [6]. The increasing use of new technologies by the population has given an important role to social networks in obtaining information on health and health promotion. Although social media is a good channel for health organizations to disseminate verified and accurate information, there is also considerable potential for misinformation that is harmful to patients [7-9]. One of the best-known social networks, Twitter, is a platform that allows short messages to be shared in real time, accompanied by images, hashtags (it serves as an indication that a piece of content relates to a specific topic or category), or mentions (ie, when the short message contains another person’s username) [10]. Twitter was created in 2006 and currently has 322.4 million monthly users around the world [11].

The increase in vaccine hesitancy, delay, and refusal despite the availability of vaccination services may be fueled, in part, by claims on the internet about the harmfulness of vaccinations [12-14]. In fact, the analysis carried out by Jamison et al [15] classifying the different topics of discussion about vaccines on Twitter suggests that there is a slightly higher proportion of antivaccine messages (22%) than provaccine messages (17%), and the remaining messages (61%) were neutral. It was identified that the main topics of antivaccine messages were security concerns and conspiracies. Conversely, provaccination users generated content promoting the vaccine, criticizing antivaccine beliefs about vaccine safety and efficacy.

Toward the end of April 2021, UNICEF and the WHO, alongside other accounts and organizations, were promoting the hashtag #VaccinesWork during World Immunization week, which took place between April 29th and 30th. Using this as a case study, the aim of this paper is to analyze the #VaccinesWork hashtag on Twitter in the context of the COVID-19 pandemic, analyzing the main messages shared and the organizations involved. To analyze the information, the study sought to address the following research questions (RQs):

- RQ1: Who were the key opinion leaders?
- RQ2: What were the most shared URLs?
- RQ3: What were the most used hashtags?

## Methods

### Data Retrieval

The data set used in this paper consists of 11,085 tweets containing the #VaccinesWork hashtag from the 29th to the 30th of April 2021. Our data set included tweet replies and mentions in tweets with the #VaccinesWork hashtag. The data were retrieved using NodeXL (Social Media Research Foundation), and the network graph was laid out using the Harel-Koren fast multiscale layout algorithm [16]. The #VaccinesWork hashtag and time period studied were selected because they corresponded to the final two days of World Immunization week. This study used the Academic Track application programming interface to retrieve tweets. The library 'academictwitteR' [17] was used to retrieve tweets. Finally, tweet IDs were entered into NodeXL. A computer running Microsoft Windows 8 was used to retrieve data in Microsoft Excel 2010 using the professional version of NodeXL (release code: +1.0.1.428+). NodeXL uses Twitter’s search application programming interface. URLs were automatically expanded within NodeXL.

### Ethics Approval

The study received ethical approval from Newcastle University, under the review number 14026/2020.

### Data Analysis

The data analysis has identified 6 network shapes and structures that Twitter topics tend to follow, such as broadcast networks, polarized crowds, brand clusters, tight crowds, community clusters, and support networks [18]. This study analyzes influential users (ie, a user who is able to reach and create debate among other users), the keywords related to the main hashtag (ie, words related to the topic that appears in the tweets), the topics (ie, the subject of which there is a thread of conversations), and web sources (ie, the URLs that people have shared by a tweet). In this study, influential users were identified by drawing upon the betweenness centrality algorithm. In addition, social network analysis of the discussion was conducted with NodeXL, as in previous research, which provided an understanding of the shape of the conversation. The graph’s vertices were grouped by cluster, using the Clauset-Newman-Moore algorithm [8,9]. Individual users have been anonymized in line with current best practices for research on Twitter [19].

## Results

### Social Network Visualization

**Figure 1:**
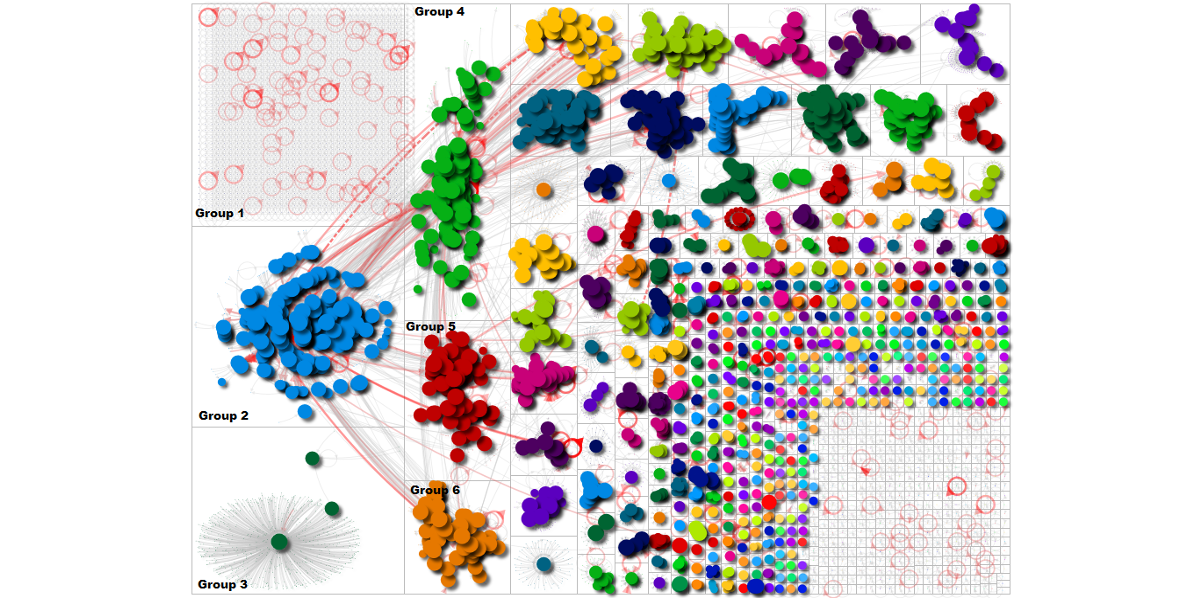
Social network graph of #VaccinesWork.

**Figure 2:**
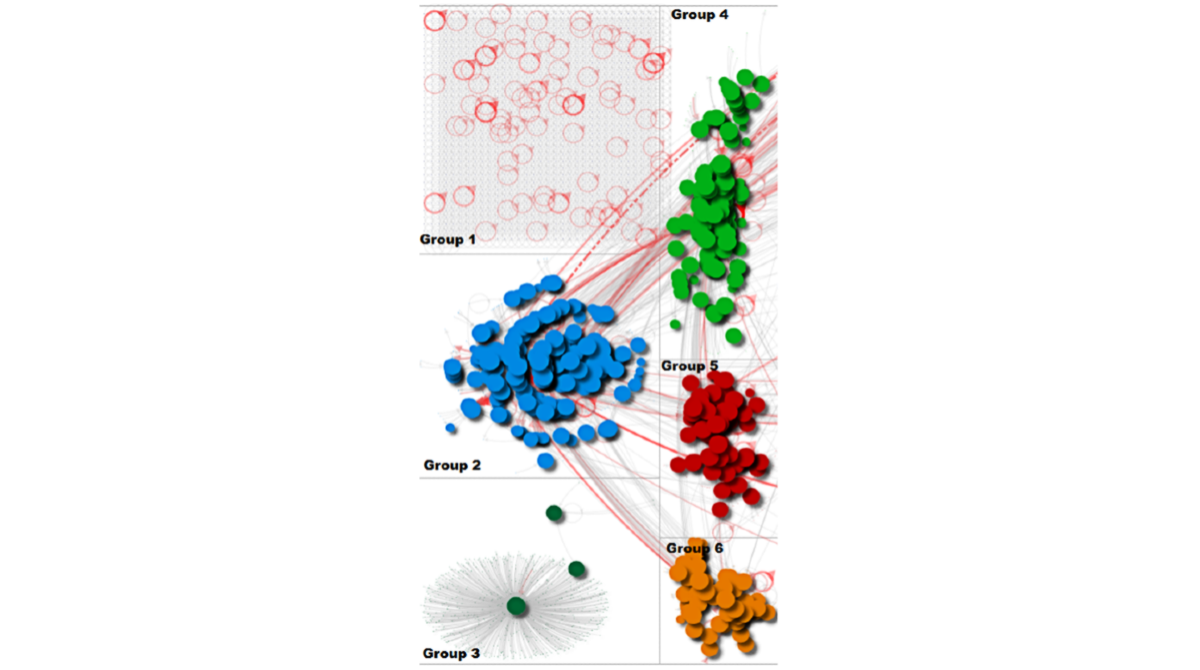
Zoomed-in social network graph of the top 6 groups.

Figure 1 provides an overview of the network and key groups of users who were tweeting using the hashtag or keyword 'VaccinesWork,' and Figure 2 provides a zoomed in view of the top 6 groups. It could be seen that groups 2, 4, 5, and 6 look like they are mentioning or retweeting each other. The circles are similar in size in these groups indicating that users in those groups were more influential. Red lines coming out of these groups indicate strong relationships with other users or groups and highlight how they have a strong influence. The graph shows various communities of users who shared and tweeted using this hashtag. The largest group in the network was of an isolated group (labelled group 1); it shows that users were tweeting without mentioning one another. They simply tweeted in support of the campaign by adding their message of support alongside ‘VaccinesWork.’ The tweet and hashtag would appear on their timeline, visible to all their followers. There were also many other smaller pockets of discussion indicating several communities that were tweeting using the hashtag.

Table 1 shows the most used hashtags across the different groups within the network. These correspond to the group labels applied to Figure 1. It can be seen that group 2 has used the hashtag #VaccinesWork the most (1315 times), followed by group 1 (1161 times). The rest are placed far away from these. Furthermore, the shape of group 3 (a broadcast network where a single user is being retweeted) demonstrates that only one main hashtag was used in this group. Between other groups, there are relevant hashtags in common promoting vaccination. ‘Covid19’ appears in second position in group 1 and group 5; in group 2 and group 4, it appears in third position.

The hashtag #worldimmunizationweek also appears across groups 1, 2, 4, and 5. In addition, there are hashtags, such as #avw2021 or #eiw2021, promoting vaccination week in Africa and Europe, respectively. Other hashtags such as #protectedtogether, #vaccinesbringuscloser, and #vaccinessavelives are related to #VaccinesWork to promote group immunity.

**Table 1:** Top hashtags in tweets per group.

Group 1	Hashtag, n	Group 2	Hashtag, n	Group 3	Hashtag, n	Group 4	Hashtag, n	Group 5	Hashtag, n
VaccinesWork	1161	VaccinesWork	1315	VaccinesWork	793	VaccinesWork	982	VaccinesWork	539
Covid19	224	Worldimmunizationweek	245	Wearamask	1	Worldimmunizationweek	312	Covid19	205
Vaccinated	150	Covid19	206	Largestvaccinedrive	1	Covid19	225	Europeanimmunizationweek	169
Vaccine	100	Avw2021	167	Vaccinated	1	Vaccinequity	196	Worldimmunizationweek	128
Getvaccinated	76	Protectedtogether	48	Stayhome	1	Dayofimmunology	63	Eiw2021	68
Worldimmunizationweek	62	Wiw2021	46	Tomandjerry	1	Healthforall	53	Worldimmunisationweek	47
Covidvaccine	52	Worldimmunisationweek	41	Doctors	1	Primaryhealthcare	52	Vaccinessavelives	37
Vaccineregistration	42	Vaccinated	38	Weremask	1	Askwho	33	Vaccination	33
Covid19vaccine	41	Endpolio	38	Indiafightscovid19	1	Eiw2021	31	Vaccinesbringuscloser	31
Covid19india	41	Wcc	38	Covidemergency2021	1	Vaccines	28	Wewontrest	29

### Most shared URLs

Table 2 provides an overview of the top 5 key URLs within tweets. The links used point to legitimate sources of information and high-quality information sources such as peer-reviewed papers.

The first most shared URL (N=136) is an article published by The Lancet. The article shared is a modelling study that estimates the health impact of vaccination against 10 pathogens in 98 low-income and middle-income countries from 2000 to 2030. The second most shared URL (N=87) is an article published by a web-based news service, available as a free-access website that provides daily and weekly newsletters to subscribers. The article is about Medicago, a pioneer company in developing plant-based vaccines and therapeutics in Canada. The company had started a rolling submission for its plant-derived adjuvanted COVID-19 vaccines candidate. The third most shared URL (N=62) belongs to the WHO. The article shows the issue of vaccine equity and the solution proposed by the organization. The fourth most shared URL (N=58) belongs to the European Vaccination Information Portal. The main purpose of this website is to provide evidence on vaccines and vaccination in general. The final most shared URL (N=45) is about the Campaign Vaccination Week in the Americas 2021 by the Pan American Health Organization.

Table 3 provides an overview of the influential users within the network. It has to be considered that betweenness centrality refers to the influence a user exerts on other users by his tweets. In addition, the concept of influence refers to the popularity or reputation of a user in the social network, calculated using the betweenness centrality metric [20]. The study identified the top 5 users who were influential based on their betweenness centrality score.

First, the betweenness centrality score ranks users among each other, such that users with higher scores have greater influence within the network; in this context, the user who has had the most influence on other users is the WHO. This is followed by UNICEF, one of the world’s largest providers of vaccines and one of the organizations that started promoting the hashtag #VaccinesWork. In this instance, the Twitter account of the WHO has a betweenness centrality score that was 70.90% greater than that of UNICEF, which was in second place. This demonstrates that the WHO has considerably more influence compared to other users.

Third place belonged to Gavi, the Vaccine Alliance, which is a public-private global health partnership with the goal of increasing access to immunization worldwide. In the fourth place was the Centres for Disease Control and Prevention (CDC) of the US Department of Health and Human Services. CDC focuses on the development and application of disease prevention and control, environmental health, and health education activities. In the fifth place was UNICEF India, in line with UNICEF’s general goal.

**Table 2:** Overview of the 5 most shared URLs.

Rank	Title	URLs	Count, n
1	Estimating the health impact of vaccination against 10 pathogens in 98 low-income and middle-income countries from 2000 to 2030: a modelling study	[21]	136
2	Plant-derived COVID-19 vaccine candidate starts rolling review with Health Canada	[22]	87
3	Call to action: vaccine equity	[23]	62
4	COVID-19 vaccines	[24]	58
5	Vaccination Week in the Americas 2021	[25]	45

**Table 3:** Overview of the top 5 influential users.

Rank	Top 5 users, ranked by betweenness centrality	User biography (date taken)	Betweenness centrality
1	WHO^a^	We are the #UnitedNations’ health agency—#HealthForAll. Always check our latest tweets on #COVID19 for updated advice/information (20/07/2021)	15285431
2	UNICEF^b^	As conflict escalates in #Ukraine, UNICEF is on the ground reaching children with water, health and education services. Here’s how you can help (20/07/2021)	8943804
3	Gavi	Gavi, the Vaccine Alliance, helps vaccinate half the world’s children against deadly and debilitating diseases. #VaccinesWork #COVAX #OneWorldProtected (20/07/2021)	3513609
4	CDC^c^global	CDC works 24/7 to save lives, reduce disease, and improve #globalhealth around the world. Links, follows, and retweets do not constitute endorsement (20/07/2021)	2650617
5	UNICEFindia	Since 1949, UNICEF has worked side-by-side with India to save children’s lives, defend their rights, and help them fulfill their potential. #ForEveryChild (20/07/2021)	2028529

^a^WHO: World Health Organization.

^b^UNICEF: United Nations International Children’s Emergency Fund.

^c^CDC: Centres for Disease Control and Prevention.

## Discussion

### Principal Findings

Social networks are part of people’s daily lives. Although Twitter is a relevant tool for obtaining verified information, conspiracy theories with incorrect information also emerge [8,9].

The hashtag #VaccinesWork was created by UNICEF in 2019 to promote immunization on social media. UNICEF ensures that every US $1 spent on childhood immunization returns up to US $44 in benefits [26]. In April 2019, the Bill & Melinda Gates Foundation contributed US $1 to UNICEF for every like or share of social media posts using the hashtag. This economic contribution to the promotion of the hashtag most probably boosted UNICEF’s early campaign. Three years after this campaign, our study analyzed the status of this hashtag after the spread of COVID-19.

To respond to RQ1, this study identified influential users who were actively tweeting and spreading information in favor of vaccines. According to Figure 1, there are many groups of users who used the #VaccinesWork hashtag. The most influential user accounts were well-known organizations, such as the WHO, UNICEF, or Gavi, among others. In this context, the results suggest that the hashtag was linked to groups of users who were tweeting factual information. The betweenness centrality metric was useful in finding users with greater influence within the network. The results highlight how influential users were effective broadcasters in favor of vaccines, and how their reach extended beyond their own network of Twitter followers, according to the number of red lines (Figure 1 and 2) that were coming out of the groups and extending to other users. They show the reach of these users. In this context, these types of social network analyses can also be useful for detecting when the volume of health-related tweets increases among the population. When the popularity of a topic increases significantly on social media, it could be the optimal moment to launch a health campaign on social and traditional media to maximize the impact [27,28].

Regarding RQ2, the most shared URL is an article published by The Lancet. This indicates that users involved with the #VaccinesWork hashtag are more likely to share information with high levels of trust. According to the article, users find the evidence provided on mortality reduction from vaccines relevant, not only in the context of COVID-19 but also concerning the mortality reduction produced by vaccines against 10 different pathogens [21]. Furthermore, regarding the most shared URLs and comparing the results with other articles about misinformation in Twitter by authors, provaccine user groups tend to disseminate articles from indexed journals [8,9]. The results have shown that the first most shared URL is from the scientific Journal The Lancet, a peer-reviewed source with a high impact factor. The other URLs are from organizations related to health issues, such as the WHO, Pan American Health Organization ,or Biopharma. In contrast, other studies on conspiracies on Twitter have shown that the most shared URLs were those of YouTube videos and press articles attempting to disprove the conspiracy [8,9].

Regarding RQ3, it is remarkable that group 3 has only promoted the hashtag #VaccinesWork, and this could suggest that this group of users may have tried to amplify this hashtag. Users who used the hashtag #VaccinesWork have other hashtags in common. #Worldimmunizationweek is a clear example of the awareness of these groups about the importance of vaccination. Overall, the hashtags related to #VaccinesWork belonged to users who promote group immunity (with hashtags such as #protectedtogether, #vaccinesbringuscloser, and #vaccinessavelives) and vaccination for other diseases and not only COVID-19, such as poliomyelitis (with hashtags such as #endpolio). There are several studies evaluating the effectiveness of health-related campaigns on social media in relation to public health. Some examples are a campaign to increase awareness of cervical cancer, a campaign promoting food safety, or a campaign to improve autism awareness [29-31]. These studies show that the effectiveness of social media campaigns depends on their ability to involve its targets. However, in the actual context of COVID-19, observing hashtags promoted by the health organizations is relevant to making recommendations for more effective campaigns related to vaccination.

### Conclusions

This study investigated the discussions surrounding the #VaccinesWork hashtag. It was found that organizations including the WHO, UNICEF, or Gavi were the key opinion leaders and had a big influence on the spread of positive and factual vaccine information among users. Social media networks containing conspiracies tend to contain dubious accounts leading the discussions and often link to unverified information. This kind of analysis can be useful to detect the optimal moment to launch health campaigns on Twitter.

## Abbreviations

CDC: Centres for Disease Control and Prevention
RQ: research question
UNICEF: United Nations International Children’s Emergency Fund
WHO: World Health Organization
